# THE INTERPLAY OF SOCIAL DETERMINANTS, DIGITAL TECHNOLOGY, AND DEPRESSIVE SYMPTOMS IN KOREAN OLDER ADULTS

**DOI:** 10.1093/geroni/igae098.3221

**Published:** 2024-12-31

**Authors:** Donghee Shin, Giyeon Kim

**Affiliations:** Chung-Ang University; Korea Disabled People’s Development Institute, Seoul, Republic of Korea; Chung-Ang University, Seoul, Republic of Korea

## Abstract

The purpose of this study was to address the mechanisms between digital inequality and mental health disparities among Korean older adults, recognizing that social determinants of health (SDH) also contribute to the digital divide. We investigate whether older adults’ digital capacity exacerbates or mitigates health inequalities in later life. Drawn from the 2020 National Survey of Older Koreans, a nationally representative sample of 9,885 adults aged 65 and older was analyzed. We employed hierarchical multiple regression, moderation, and mediation analyses. SDH factors including sex, income, education and geographic location (rural-urban residence) were examined. Results showed that capacity to use digital technology significantly moderates and mediates the relationship between income, education, geographic location and depressive symptoms. Notably, digital technology use buffers against the negative effects of low income (B=.021, p<.05) and living in metropolitan areas (B=.095, p<.001, B=.067, p<.01) on depressive symptoms, while it also amplifies the negative effect of low educational attainment on depressive symptoms (B=-.030, p<.001). Specifically, heightened digital technology use among older adults augments the detrimental effects of education on depressive symptoms. Findings suggest that the importance of developing diverse support programs to improve digital literacy among Korean older adults, offering possible ways for reducing the digital divide and addressing mental health disparities.

